# Recognition and Management of Acute Purpura Fulminans: A Case Report of a Complication of Neisseria meningitidis Bacteremia

**DOI:** 10.7759/cureus.13704

**Published:** 2021-03-04

**Authors:** Michael C Kim, Jaimin Patel

**Affiliations:** 1 Department of Medicine, Graduate Medical Education, Methodist Dallas Medical Center, Dallas, USA

**Keywords:** purpura fulminans, neisseria meningitidis, coagulation cascade, ivig, tissue necrosis

## Abstract

Purpura fulminans (PF) is a rare, potentially fatal complication of disseminated intravascular coagulation that is commonly associated with severe bacterial infections such as those caused by the bacterium *Neisseria meningitidis*. With the advent of vaccination, meningococcal disease has become infrequent, with a reported incidence of 1 case per 100,000 people per year. PF is an even rarer phenomenon that is only found in approximately 10 to 20% of patients with meningococcal septicemia. PF can cause irreversible tissue necrosis within 48 hours and, in severe cases, death. Early recognition is crucial as PF has a mortality rate as high as 60% in patients with meningococcal disease. Prompt recognition, treatment of the underlying cause, vigorous skin care, and multispecialty collaboration are required for optimal management of PF, though morbidity and mortality remain high as there is no cure for adult PF. We present a case of acute PF in a patient who presented with septic shock secondary to *Neisseria *bacteremia.

## Introduction

Purpura fulminans (PF) is a rare complication of disseminated intravascular coagulation (DIC). DIC is caused by dysregulation of the coagulation cascade, and can lead to intravascular thrombosis and irreversible tissue necrosis [1,2]. PF is considered a hematological and dermatological emergency in which hereditary or neonatal PF occurs in 1 out of 1,000,000 neonatal populations and acute septic PF occurs in only 10 to 20% of patients with meningococcal septicemia [3]. It is a rare phenomenon, but PF can vary depending on etiologies and risk factors. Acute septic PF most commonly occurs in the adult population in the setting of severe bacterial infection where endotoxins and inflammatory cytokines cause consumption of anticoagulants such as antithrombin II, protein C, and protein S, which leads to coagulation cascade dysregulation and subsequently to skin necrosis [4]. Once PF develops, progression to irreversible tissue damage and potential death from septic shock and multiorgan failure can occur within 48 hours, so prompt recognition, treatment of the underlying cause, vigorous skin care, and multispecialty collaboration are needed [1]. We present a case of acute PF in an otherwise healthy young male patient who presented with septic shock secondary to *Neisseria* bacteremia.

## Case presentation

A 32-year-old African American male with no significant past medical history presented to the emergency department with sudden onset bilateral foot pain that had progressed up his legs. The pain started a few days prior to presentation without any inciting events. He reported that the pain was a 6 to 7 out of 10 in severity, burning in nature, exacerbated by movement, and he denied any alleviating factors. He reported a fever of 39.4 °C at home one day prior to arrival as well as nausea, vomiting, dyspnea, cough, and generalized myalgias. He otherwise denied any headache, pain with neck flexion, abdominal pain, dysuria, diarrhea, weakness or numbness of extremities, recent illness, or sick contacts. Patient did not have any pertinent family medical history and denied any illicit drug use other than marijuana. 

On initial evaluation, the patient’s vitals were notable for a temperature of 37.1 °C, heart rate of 126 beats per minute, respiratory rate of 23 breaths per minute, and blood pressure of 90/52 mmHg. Physical exam revealed grossly benign cardiovascular, abdominal, and dermatologic exams without signs of erythema or rash on the patient's body including face, bilateral upper and lower extremities, and dorsal and ventral surfaces of the upper body. Patient, however, endorsed pain to palpation on his bilateral lower extremities. Physical exam was also negative for nuchal rigidity, Kernig sign, and Brudzinski sign. Labs were notable for a low white blood cell count of 1.7 x 103/µL (reference range: 4.0 x 103 to 11.0 x 103/µL) and an elevated lactic acid level of 5.5 mmol/L (reference range: 0.5 to 2.2 mmol/L). Patient also tested negative for coronavirus disease 2019 (COVID-19) antigen, COVID-19 polymerase chain reaction (PCR), and HIV. Chest and abdominal imaging were benign, and ultrasound of the bilateral lower extremities was negative for deep vein thrombosis.

The patient was determined to be in septic shock from an unknown source. As his blood pressure failed to improve after receiving 5L of IV fluids, he was admitted to the intensive care unit (ICU) and was started on norepinephrine, vasopressin, phenylephrine, and dexamethasone along with empiric antibiotics vancomycin and cefepime. His ICU course was complicated by DIC characterized by low platelet count (<45 x 103/µL; reference range: 150 x 103 to 450 x 103/µL), low haptoglobin (<20 mg/dL; reference range: 50-220 mg/dL), elevated lactic acid dehydrogenase (848 U/L; reference range: 140-280 U/L), increased prothrombin time (PT) (20.0 seconds; reference range: 11.3-14.7 seconds), partial thromboplastin time (PTT) (34 seconds; reference range 23-37 seconds), elevated international normalized ratio (INR) (1.6; reference range: 0.9-1.2) and elevated D-dimer (>20.0 ug/mL fibrinogen-equivalent units [FEU]; reference range: ≤0.5 µg/mL FEU), which required transfusion of two units of platelets for DIC as the patient’s platelet count continued to drop after each transfusion. Blood cultures came back positive for *Neisseria meningitidis* and cerebrospinal fluid studies were negative for infection. To treat the *Neisseria* bacteremia, the patient’s antibiotic regimen was changed to ceftriaxone 2 g IV twice per day for two weeks.

**Figure 1:**
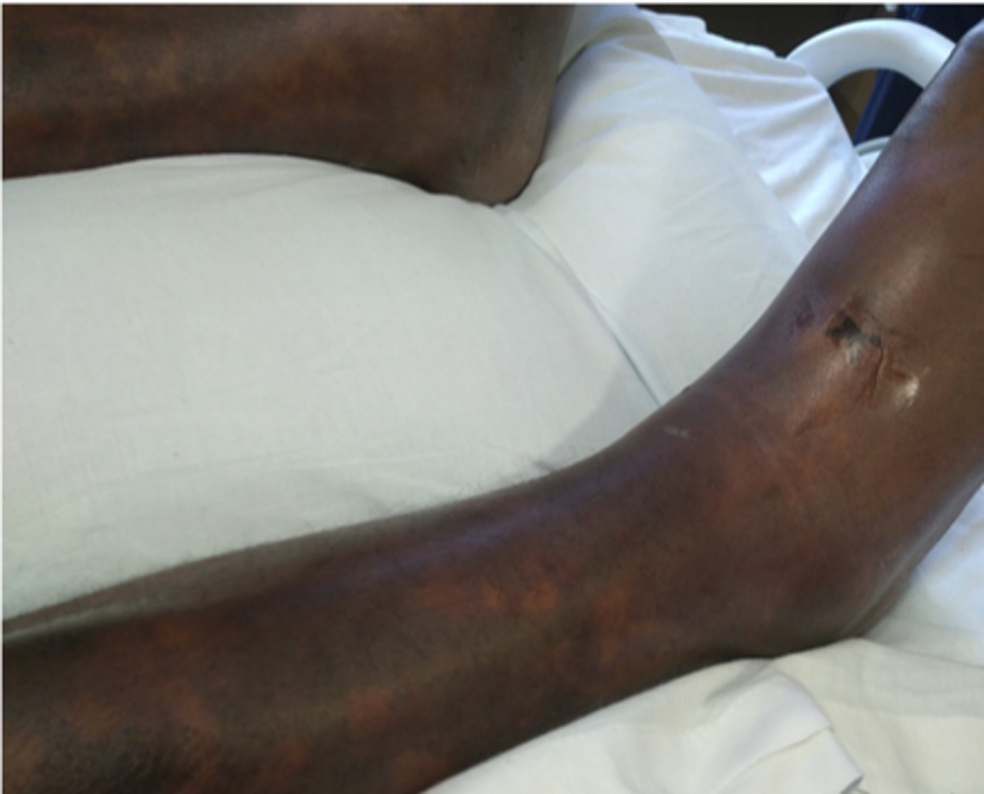
Initial (October 21, 2020) presentation of Purpura Fulminans.

The patient’s clinical status improved, but after being transferred to the medicine wards service on day four of his hospitalization, he endorsed worsening bilateral lower extremity pain, and a rapidly worsening rash that was present two days prior to being transferred out of the ICU. On examination, the patient’s distal lower extremities showed a large irregular macular purpuric rash with an irregular pattern and pain out of proportion (Figure 1). Other than distal lower extremities, there were no signs of macular purpuric rash anywhere else on the patient’s body including face, upper extremities, dorsal and ventral surfaces of the upper body, as well as bilateral proximal lower extremities.

**Figure 2:**
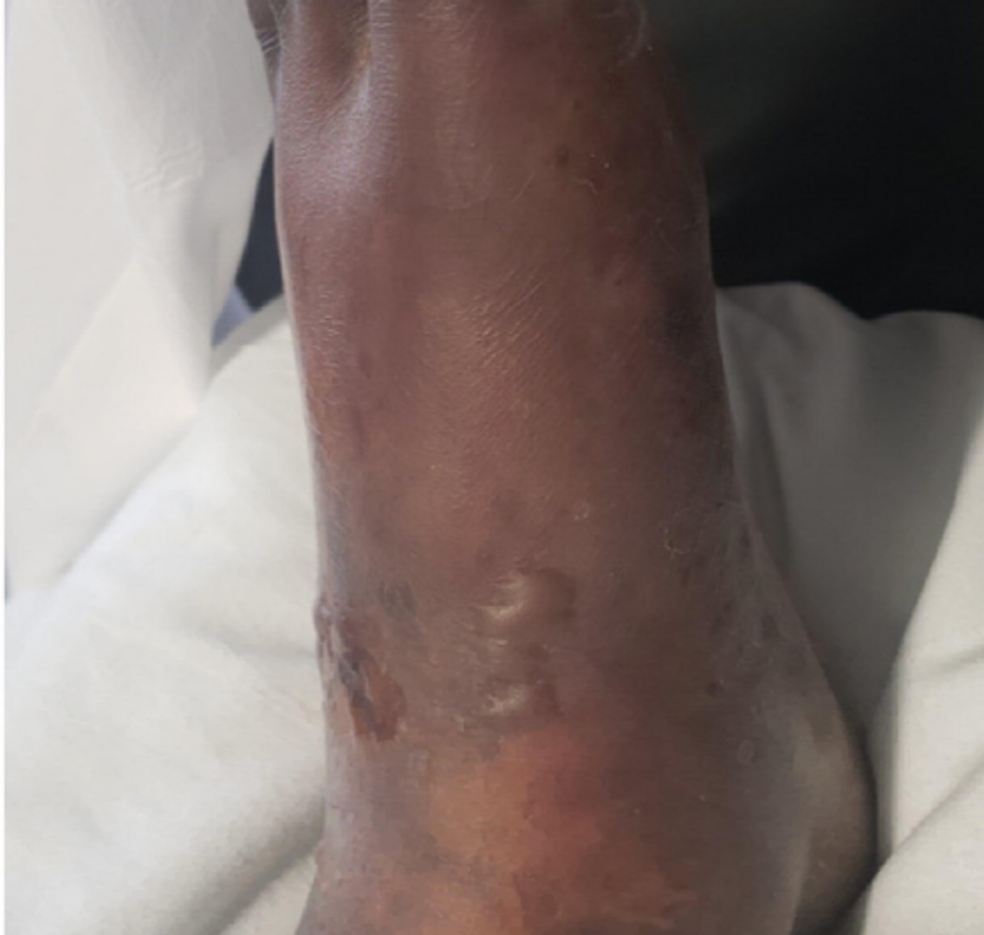
Formation of blisters after intravenous immunoglobulin (IVIG) administration.

**Figure 3:**
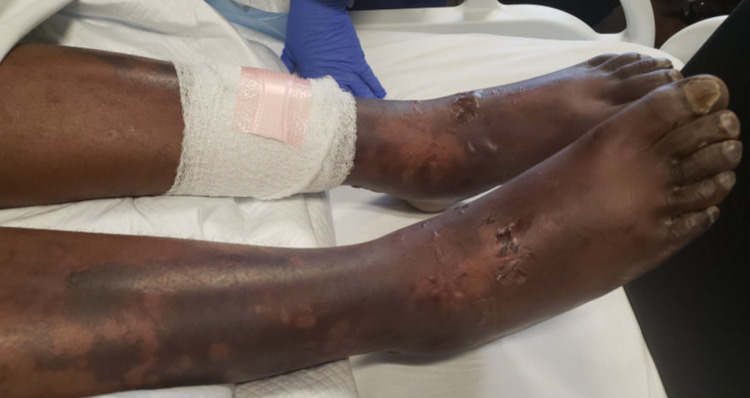
Clinically stable appearing Purpura Fulminans after intravenous immunoglobulin (IVIG) treatment.

The patient’s presentation was concerning for necrotizing fasciitis, compartment syndrome, or PF. Surgery was consulted, and compartment syndrome and necrotizing fasciitis were ruled out based on the clinical exam and bedside tissue dissection which revealed viable healthy-appearing subcutaneous tissue and fascia. Given the patient’s clinical history and exam (i.e., rash and *Neisseria* bacteremia), it was determined by consensus amongst the primary, surgery, dermatology, and infectious disease teams that the patient was likely suffering from PF. The patient was given IV fluids, a 10% infusion of intravenous immunoglobulin (IVIG), adequate pain management, and wound care consultation for close monitoring of his lower extremities for possible progression to gangrene. After administration of IVIG, small blisters started to form, but the patient expressed lessening pain and the rate of rash progression seemed to decrease (Figures 2, 3).

**Figure 4:**
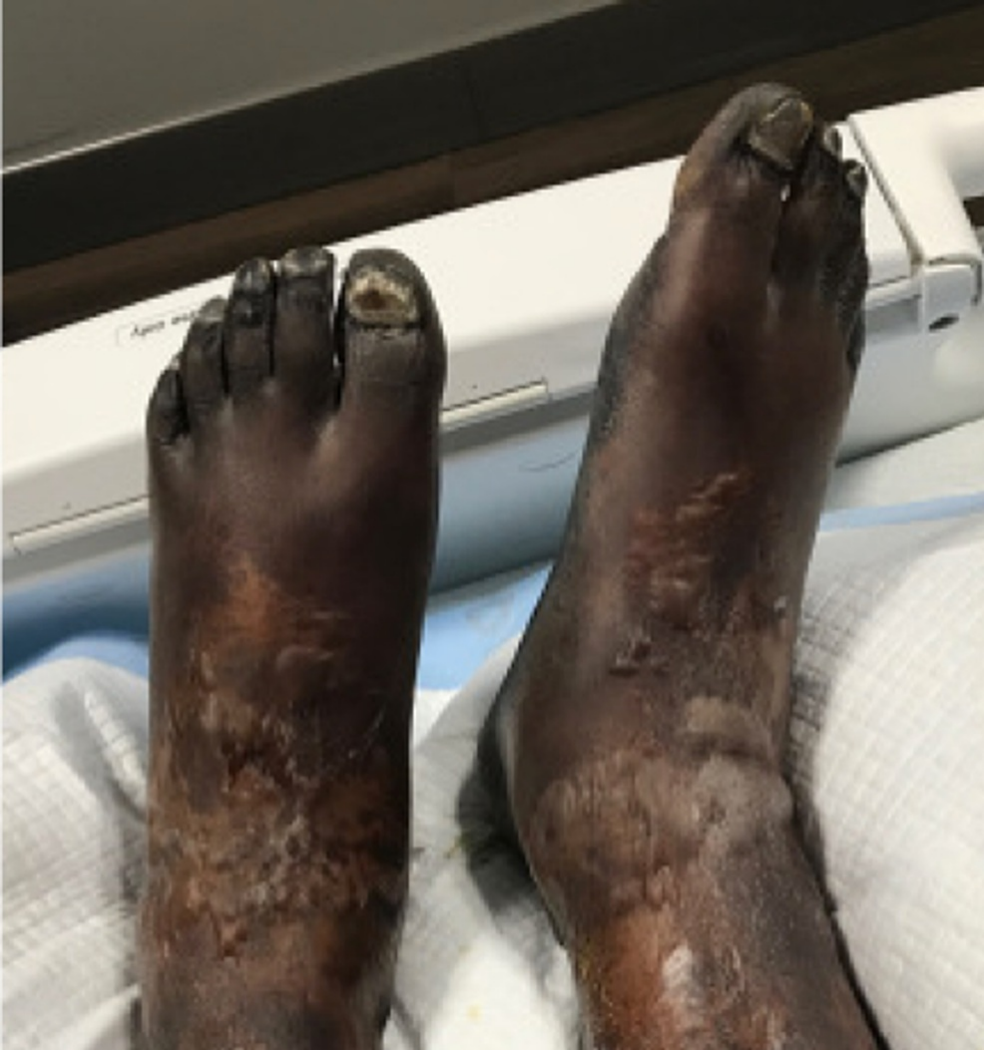
Bilateral gangrene on all 10 digits of the feet (October 28, 2020).

On day 12 of hospitalization, gangrene of all 10 digits of the feet was apparent (Figure 4). Arterial Doppler ultrasound of the lower extremities did not show any major occlusive disease. Podiatry planned for bilateral amputation of all 10 digits of the feet, but intraoperative fluorescent angiography revealed lack of viable blood perfusion from the midfoot to the toes. Podiatry determined that due to lack of viable perfusion, wound will not heal properly if the patient underwent toe amputations, so the patient ultimately underwent bilateral below the knee amputations by vascular surgery on day 19. The patient tolerated the surgery well, and he was eventually discharged on day 23 of hospitalization in stable condition with a wheelchair and continued outpatient physical therapy.

## Discussion

PF is considered to be a manifestation of certain systemic illnesses rather than a disease itself. First described by Guelliot in 1884 among pediatric patients in the setting of bacterial or viral infections, PF is associated with various clinical scenarios, but it is commonly associated with severe infections [1].

PF commonly demonstrates bimodal age distribution at diagnosis in pediatric populations from ages one to three years old and adolescents from 16 to 18 years old [1]. PF in adult populations is a rare phenomenon in which hereditary or neonatal PF occurs in 1 out of 1,000,000 neonatal populations and acute septic PF occurs in only 10 to 20% of patients with meningococcal disease [3]. With the advent of vaccination, the incidence of meningococcal disease has decreased to less than 1 case per 100,000 per year in the United States; among these cases approximately 15 to 25% develop PF [1,5]. *N. meningitidis* and *Streptococcus pneumoniae* are the most common bacteria associated with PF, but *Capnocytophaga canimorsus*, *Haemophilus influenzae*, and *Staphylococcus aureus* can also be associated with PF [1]. Development of PF in meningococcal disease is a poor prognostic indicator with a mortality rate of anywhere from 20 to 60%, whereas meningococcal disease without PF has a mortality rate of 15%.

PF is believed to be the result of anticoagulation mechanism dysfunction, particularly involving protein C [1]. In the setting of severe bacterial infection, PF may develop secondary to DIC where anticoagulants such as protein C, protein S, and antithrombin III are depleted [1]. This pro-coagulable state can lead to multisystemic thrombosis and multiorgan failure, which ultimately leads to death.

PF can be categorized as acute septic PF, post-infectious or idiopathic PF, and hereditary or neonatal PF [2,6]. Acute septic PF occurs in the setting of bacterial infection, and can be accompanied by septic shock and multiorgan failure [6]. Lesions generally involve distal extremities, and progress rapidly in an ascending fashion [6]. It is believed that during infection, bacterial endotoxin and inflammatory cytokines such as interleukin (IL)-12, IL-1, interferon gamma (IFNγ), and tumor necrosis factor alpha (TNFα) cause consumption of anticoagulants such as antithrombin III, protein C, and protein S [4]. The consumption of anticoagulants leads to dysregulation of the coagulation cascade and subsequently to tissue necrosis.

Compared to acute septic PF, idiopathic PF is thought to be caused by immunoglobulin G (IgG) autoantibodies against protein C or protein S [1]. Common pathogens associated with idiopathic PF are varicella and Streptococcal species, and development usually occurs seven to 10 days after infection where lesions tend to develop in the buttocks and lower extremities while sparing the distal extremities [1].

Lastly, neonatal PF is found in neonates with severe protein C deficiency, and in rare cases, protein S deficiency [1,2]. Development of PF can be rapid, where progression from skin necrosis to gangrene of lower extremities and genitalia occurs within matter of few hours to days after birth [1]. Also, these pediatric patients tend to develop cerebral venous thrombosis which leads to severe neurologic symptoms and even multiorgan failure [1,6]. 

To promptly diagnose PF, one must fully understand a patient’s clinical history and laboratory values as well as the context in which PF developed. On physical examination, PF generally manifests with cutaneous pain that is followed by erythema and petechiae on either the trunk, extremities, or both [1,7]. The petechiae then coalesce into irregularly shaped areas of hemorrhagic necrosis with a blue-black color; bullae or vesicles may also form in certain cases [7]. The evolution of PF can occur within 24 to 48 hours, leading to irreversible necrosis of the skin, multiorgan failure, and death [1].

When evaluating for PF, laboratory workup should involve a complete blood count, a chemistry panel, and a coagulation panel. As PF represents a dysregulation of hemostasis, PF generally has laboratory results consistent with DIC, which includes prolonged coagulation times (prothrombin time and partial thromboplastin time), elevated D-dimer, decreased fibrinogen, and thrombocytopenia [1]. Also, PF can appear similar to necrotizing fasciitis, so a laboratory risk indicator for necrotizing fasciitis (LRINEC) score should be calculated if there is a low index of suspicion for PF [3].

Over the years, various treatment methods have been tested for management of neonatal PF, but a definitive treatment guideline or therapy for acute septic PF has yet to be determined. Therefore, management involves treatment of underlying cause and supportive measures. Treatment of infection includes broad-spectrum antibiotics such as vancomycin and piperacillin-tazobactam that cover *N. meningitidis*, *Streptoccoccus*, *Staphyloccocus*, and *Clostridia* species (3). Adequate fluid resuscitation and supportive care with vasopressors are needed as well when clinically indicated. Autoantibodies against protein C or protein S may have roles in PF. Therefore, immunomodulators such as corticosteroids may have theoretical benefits, but there has yet to be a definitive recommendation due to lack of sufficient data [3].

Other therapies such as hyperbaric oxygen therapy, fresh frozen plasma, protein C concentrate, IVIG, and plasma exchange have been discussed for PF management in acute septic PF, but there is no clear guideline due to inadequate data [5]. As endotoxin is one of the factors that affect the coagulation cascade in acute septic PF, IVIG putatively binds to bacterial endotoxin and neutralizes its effects [3,8]. However, a randomized, double-blind trial of 653 patients diagnosed with sepsis showed that 28-day mortality was comparable between patients receiving IVIG and placebo [9]. Dosing parameters for IVIG have been determined in acute septic PF patients: dose ranges from 400 to 600 mg/kg with a starting infusion rate of 0.01 mL/kg/min, and increasing the rate every 30 minutes until it reaches a maximum of 0.08 mL/kg/min or 8 mg/kg/min if 10% solutions are used [10]. However, IVIG, has been associated with aseptic meningitis and thromboembolic events, so risks and benefits must be weighed prior to initiation. In our patient, one dose of 30g of 10% infusion IVIG was given with rate of 8 mg/kg/min, and signs complications were closely monitored.

As mentioned above, protein C consumption during severe infection can lead to tissue necrosis in PF. Protein C concentrate putatively has positive effects in acute septic PF treatment. Several trials have attempted to evaluate the efficacy of protein C concentrate, but due to limited sample sizes and weakness in study designs, no definitive recommendations have been made [1]. A few case reports have shown that therapeutic plasma exchange in patients with severe sepsis (with or without PF) stabilizes hemodynamics, decreases IL-6 and TNF-α levels, and improves skin lesions [1]. However, due to a lack of clinical trials examining therapeutic plasma exchange in PF patients, it is difficult to evaluate whether therapeutic plasma exchange is a viable treatment option for PF [1].

First discovered in 1887, *N. meningitidis* is associated with many infections. As many as 10% of the population are carrying the bacteria in their nose and throat as asymptomatic carriers [5]. As mentioned above, meningococcal disease and acute septic PF are extremely rare phenomena in the United States due to the advent of vaccination. Our patient lacked any known history of hereditary anticoagulant deficiency or immunocompromising pathologies and was up-to-date on his vaccinations. The patient had several sexual partners and was incarcerated several months prior to his hospitalization, but the patient was overall healthy without any past medical history and his test results were negative for HIV, COVID-19 antigen, and COVID-19 PCR.

**Figure 5:**
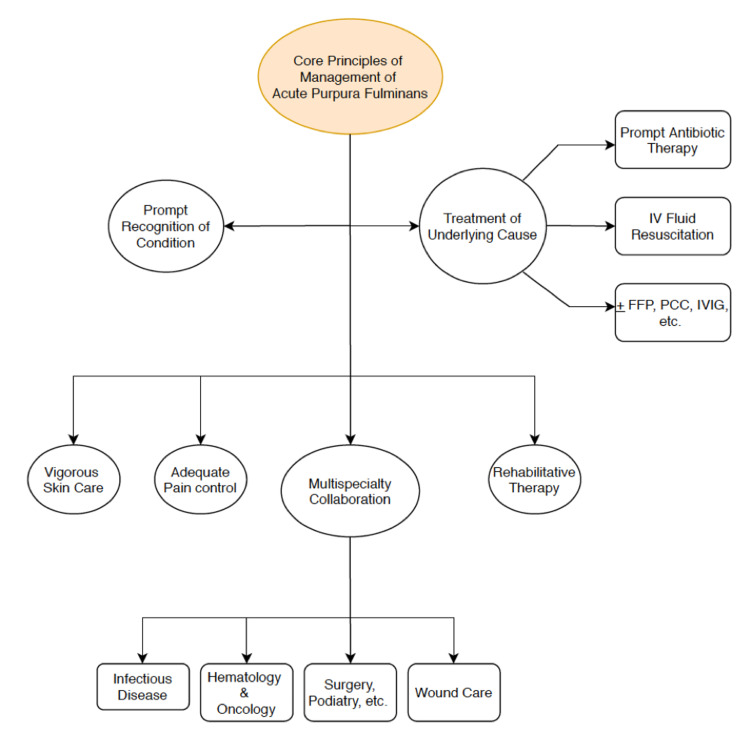
Core principles of acute Purpura Fulminans management. Abbreviations: IV – Intravenous; FFP – Fresh frozen plasma; PCC – Protein C concentrate; IVIG – Intravenous Immunoglobulin

Although PF in adult populations currently lacks definitive treatment guidelines, there are core principles that must be followed for optimal patient care: prompt recognition of the condition, treatment of the underlying cause, adequate pain control, vigorous skin care, and rehabilitative therapy (Figure 5). Multispecialty collaboration involving various specialties such as infectious diseases, hematology, wound care, surgery, and podiatry is crucial in PF management. Assistance from infectious diseases and hematology are highly valuable when making decisions to administer therapies such as IVIG or protein C concentrate. IVIG was administered to our patient, as both infectious diseases and hematology had more experience with IVIG as opposed to protein C concentrate, and because it was believed that the patient’s PF was most likely caused by coagulation cascade dysregulation from bacterial endotoxin. The collaborative effort from different medical subspecialties helped us to make a decision in a timely manner to treat the PF, which led to stabilization in the patient’s condition.

Vigorous skin care is another important component of PF management. As PF progresses, bullae or vesicles may form in certain cases. Vesicles can progressively enlarge and may burst, which can become another source of infection, so good and frequent wound care is needed. In our patient, administration of IVIG appeared to halt the tissue necrosis, but bullae and vesicles began to form. In order to reduce the risk of soft tissue infection, wound care was involved in the patient’s care as soon as he was diagnosed with PF.

PF can lead to irreversible tissue damage that can lead to gangrene, so surgical specialties must be involved in patient care. In our patient, as soon as it was recognized that the patient’s toes were becoming gangrenous, podiatry and vascular surgery were immediately consulted which led to prompt surgery before the patient’s condition worsened.

## Conclusions

PF is a rare complication of DIC in the setting of severe infection with potential to cause irreversible damage and even death. Compared to neonatal PF, there is not enough evidence-based research or recommendations for treatment and management of acute septic PF. Various treatment methods have been tested in the past, but further studies need to be done for more definitive treatment guidelines. Despite the lack of guidelines, physicians can still provide optimal care and even save lives by following core principles that include prompt recognition of PF, treatment of the underlying cause, adequate pain control, vigorous skin care, multispecialty collaboration, and rehabilitative therapy.
